# Research trends and performance of endothelin A receptor antagonist in kidney care: a bibliometric analysis

**DOI:** 10.1080/0886022X.2025.2487212

**Published:** 2025-04-11

**Authors:** Wisit Cheungpasitporn, Pajaree Krisanapan, Supawadee Suppadungsuk, Charat Thongprayoon, Tibor Fülöp, Jing Miao, Karim M. Soliman, Yuh-Shan Ho

**Affiliations:** a Department of Medicine, Division of Nephrology, Mayo Clinic, Rochester, MN, USA; b Department of Nephrology, Department of Internal Medicine, Thammasat University, Khlong Nueng, Thailand; c Faculty of Medicine Ramathibodi Hospital, Chakri Naruebodindra Medical Institute, Bang Pla, Thailand; d Medical Services, Ralph H. Johnson VA Medical Center, Charleston, SC, USA; e Department of Medicine, Division of Nephrology, Medical University of South Carolina, Charleston, SC, USA; f Trend Research Centre, Asia University, Taichung, Taiwan

**Keywords:** Endothelin A receptor antagonists, hypertension, chronic kidney disease, diabetic nephropathy, focal segmental glomerulosclerosis, bibliometric analysis

## Abstract

**Background:**

Endothelin A receptor antagonists (ERAs) have emerged as pivotal therapeutic agents in managing pulmonary hypertension (PH) and various kidney disorders, including chronic kidney disease (CKD) and proteinuric glomerular diseases such as IgA nephropathy (IgAN) and focal segmental glomerulosclerosis (FSGS). Although initially developed for pulmonary applications, recent research has highlighted their renoprotective effects, expanding their role in nephrology. This study presents a comprehensive bibliometric analysis of global research trends, key contributors, and emerging applications of ERAs in kidney care over the past three decades.

**Methods:**

A bibliometric analysis was performed using the Science Citation Index Expanded database (1992–2023). Relevant kidney-related publications were identified through specific keyword searches. Author performance was assessed using the *Y*-index.

**Results:**

ERA-related research has shown significant growth, particularly in nephrology. The United States and the University of Groningen lead in publication volume and international collaborations, with H.J.L. Heerspink emerging as a key contributor. While PH remains the dominant research focus, nephrology applications are rapidly increasing, particularly in CKD, diabetic nephropathy (DN), and glomerular diseases. A major milestone was the accelerated FDA approval of sparsentan for IgAN in 2023, followed by full approval in 2024 based on confirmatory efficacy data. However, challenges such as fluid retention and cardiovascular risks remain, necessitating further investigation into optimized ERA therapies, including combination strategies with SGLT2 inhibitors.

**Conclusions:**

The expanding role of ERAs in nephrology underscores their potential in treating proteinuric kidney diseases. Ongoing international collaborations are advancing research on ERA safety, efficacy, and novel therapeutic strategies, supporting their broader clinical application.

## Introduction

Endothelin A (ETA) receptor antagonists have emerged as significant therapeutic agents in the management of various diseases, including those affecting the cardiovascular system and the kidneys [1–6]. Their importance has been steadily increasing due to their role in managing conditions such as pulmonary hypertension (PH) and chronic kidney disease (CKD), both of which have significant impacts on global health [4,6–8].

PH is characterized by high blood pressure in the pulmonary arteries, leading to right heart failure and a reduced life expectancy. Globally, PH affects approximately 1% of the global population, with a higher prevalence among older adults [9]. Within the spectrum of PH, pulmonary arterial hypertension (PAH) has been the primary focus, given the central role of the endothelin pathway in its pathogenesis [3,10,11]. ETA receptor antagonists (ERAs) have been essential in managing PAH by inhibiting this pathway, thus offering therapeutic benefits [2,4,7,12,13].

ETA receptor antagonists have shown significant potential in treating kidney diseases, particularly those that lead to CKD and ESKD [14,15]. The involvement of the endothelin system, and especially ETA activation, in the progression of CKD is well-documented. It contributes to various pathological processes, including vasoconstriction of afferent arterioles, inflammation, and fibrosis [16–18]. ETA receptor antagonists have demonstrated their ability to mitigate these effects, thereby offering a promising therapeutic strategy for CKD management [14,17].

The significance of targeting the endothelin system extends to glomerular diseases such as focal segmental glomerulosclerosis (FSGS) and IgA nephropathy (IgAN), which are key contributors to proteinuria and the progression of CKD [6,15,19–21]. Studies have suggested that the antiproteinuric effect of ERAs can be substantial, even when added to renin–angiotensin system (RAS) inhibition, which is a standard treatment approach for these conditions [6,20,22–24].

This study’s necessity stems from the dynamic and evolving landscape of research concerning ERAs. A detailed bibliometric analysis offers a panoramic view of the research trends, performances, collaborations, and contributions from a global perspective. It aims to quantify the scientific output, identify influential researchers and institutions, and map the thematic focuses over the past three decades.

## Data sources and methods

The collection of data for this research was conducted on 1 February 2024, involving the extraction of data from the online repositories of the Science Citation Index Expanded (SCI-EXPANDED). Based on the most recent Journal Citation Reports (JCRs) published by Clarivate on 28 June 2023, a total of 9637 journals are listed across 178 categories in the Web of Science segments within SCI-EXPANDED.

To secure comprehensive coverage during the search, strategies included the application of quotation marks and the Boolean operator ‘or.’ This approach ensured the inclusion of at least one of the designated search terms in the TOPIC field, which encompasses the title, abstract, author keywords, and Keywords Plus, spanning publications from 1992 to 2023. The selection of search terms focused on ‘Endothelin A receptor antagonist’ incorporated a variety of terms such as: ‘Endothelin A receptor antagonist,’ ‘Endothelin A receptor antagonists,’ ‘endothelin A (ETA) receptor antagonist,’ ‘ETA receptor antagonist,’ ‘ETA receptor antagonists,’ ‘endothelin ETA receptor antagonist,’ ‘endothelin ETA receptor antagonists,’ ‘Ambrisentan,’ ‘Aprocitentan,’ ‘Atrasentan,’ ‘Avosentan,’ ‘Clazosentan,’ ‘Sitaxsentan,’ and ‘Sparsentan.’ Furthermore, search terms related to ‘kidney’ included: ‘kidney failure,’ ‘kidney disease,’ ‘kidney diseases,’ ‘renal disease,’ ‘renal diseases,’ ‘renal dysfunction,’ ‘hypertension,’ ‘hypertensive,’ ‘nephropathy,’ ‘nephropathies,’ ‘glomerular disease,’ ‘glomerular diseases,’ ‘glomerulonephritis,’ ‘glomerulopathy,’ ‘glomerulopathies,’ ‘glomerulosclerosis,’ ‘dialysis,’ ‘hemodialysis,’ ‘proteinuria,’ ‘albuminuria,’ ‘microalbuminuria,’ ‘diabetic nephropathies,’ and ‘diabetic nephropathy.’ The analysis focused on articles that traditionally include sections on introduction, methods, results and discussions, and conclusions. In total, 1028 articles featuring the specified search terms within the topic area were identified in the SCI-EXPANDED database.

In 2011, the ‘front page’ including title, abstract, and author keywords as a filter was proposed to improve search strategy when used the terms of Topic (TS) in the Web of Science Core Collection for the bibliometric study [25]. The ‘front page’ filter can avoid introducing unrelated publications for bibliometric analysis. In recent years, a significant difference was found in wide range of bibliometric studies using the ‘front page’ as a filter, for example, research medical topics: breast reconstruction [26], Q fever [27], and temporomandibular disorders [28].

A total of 879 (85.5%) of the 1,028 articles containing at least one of the search keywords about ‘endothelin A receptor antagonist’ and 858 (83.5%) of the 1,028 articles containing at least one of the search keywords about ‘kidney failure’ in their ‘front page.’ Finally, 713 articles (69.4% of the 1028 articles) containing the search keywords in their ‘front page’ were defined as ERA in kidney care articles in the SCI-EXPANDED.

The comprehensive datasets from SCI-EXPANDED, including yearly citation metrics, were transferred to Microsoft 365 Excel (Redmond, WA) for further analysis. To refine the analytical process, manual coding practices were applied [29,30]. Impact factors for the journals for the year 2022 were sourced from the JCRs issued in 2022.

For the purpose of achieving more nuanced and precise insights from the scientific research analysis, this investigation adopted an advanced categorization method that took into account the corresponding authors, their affiliated institutions, and the countries of origin. Although the SCI-EXPANDED database typically assigns the role of the corresponding author to the reprint author, this study chose to consistently use the term ‘corresponding author’ for clarity [31]. In instances where articles within the Web of Science Core Collection did not specify these details, a default categorization was applied, identifying a singular author, institution, and country to serve dual roles as both the initial and corresponding entities. In multi-corresponding author articles, all corresponding authors, institutions, and countries were considered separately [28]. Articles with corresponding authors in SCI-EXPANDED, that had only address but not affiliation names were checked out and the addresses were changed to be affiliation names [28]. Finally, affiliations from England, Scotland, North Ireland (Northern Ireland), and Wales were reclassified as being from the United Kingdom (UK) [32].

In the assessment of the publication output from various countries and institutions, this study employed five publication metrics as suggested by Wang et al. [33]:

*TP*: represents the aggregate count of publications
*IP*: denotes the quantity of publications authored exclusively by entities from one country (*IP*_C_) or a singular institution (*IP*_I_)
*CP*: signifies the tally of publications resulting from international collaborations (*CP*_C_) or collaborations between different institutions (*CP*_I_)
*FP*: number of first-author articles
*RP*: number of corresponding-author articles

*Y*-index was used to evaluate publication performance of authors. The *Y*-index is defined as [34,35]

*Y*-index (*j*, *h*)

In this model, *j* represents a constant reflecting the publication potential, encapsulated by the aggregate of articles where the author is either the first or the corresponding author. The variable *h*, on the other hand, is a constant indicative of the publication dynamics, described by the polar angle representing the ratio of RP (corresponding-author articles) to FP (first-author articles). A higher *j* value suggests a more significant contribution by the author as either the first or corresponding author across their publications.

The condition *h* = π/2 is associated with authors whose publication portfolio consists solely of corresponding-author articles, where *j* quantifies these corresponding-author articles. An angle satisfying π/2 > *h* > π/4 denotes authors with a predominance of corresponding-author articles over first-author articles (*FP* > 0).

When *h* = π/4, it implies an equilibrium between first-author and corresponding-author articles, indicating a balanced contribution in both capacities (*FP* > 0 and *RP* > 0). Conversely, a condition where π/4 > *h* > 0 reflects a scenario with more first-author articles than corresponding-author articles (*RP* > 0).

Finally, *h* = 0 signifies authors whose scholarly output exclusively comprises first-author articles, with *j* representing the count of these first-author publications.

## Results and discussion

### Language of publication

The language of publication is one of the basic concerns in bibliometric studies as a big data analysis [25]. A total of 695 articles (97% of the 713 articles) were published in English, followed distantly by German (eight articles), Russian [4], Spanish [3], French [2], and Polish [1]. Non-English articles had a lower average number of authors per publication (*APP*) with 3.7 authors. The *APP* of English articles was 7.0 authors with the maximal number of 33 authors in an article.

### Publication outputs

**Figure 1.**
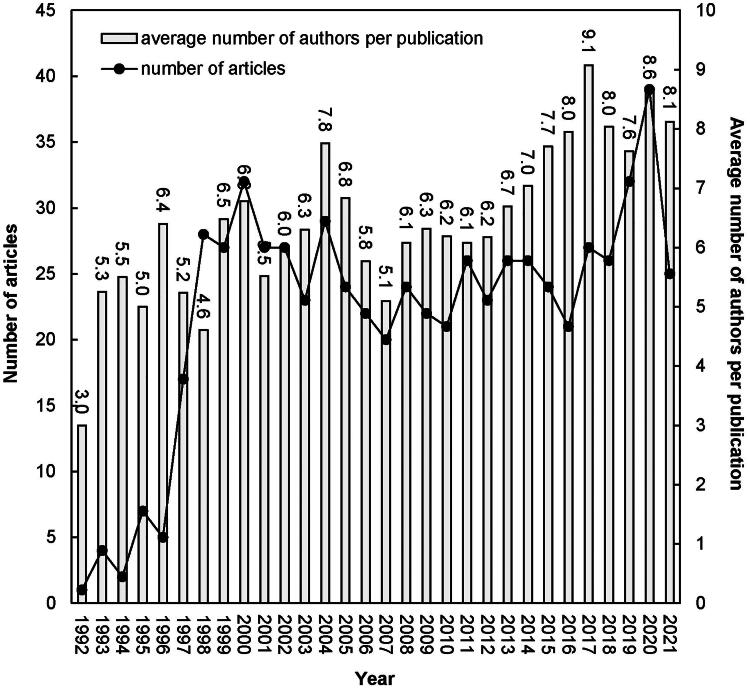
Number of articles by year.

Figure 1 provides a comprehensive view of the publication trends related to ERA research. It highlights the fluctuating interest and collaborative efforts in the field from 1989 to the present. The figure suggests an initial surge in research output from 1989 to 1993, which may have been inspired by groundbreaking studies that introduced endothelin receptor antagonists, such as bosentan, for hypertension management. One such influential study by Krum et al. [11], demonstrated the effectiveness of these antagonists in lowering blood pressure, setting the stage for increased research activity observed in the late 1990s.

The subsequent increase in publications post-2009 coincides with expanded research into the renal effects of endothelin, particularly due to Dhaun et al. [36], which explored endothelin-1’s (ET-1) role in kidney function and diseases. This period marks a significant shift toward investigating the therapeutic potential of endothelin antagonists in renal pathologies.

The increased collaborative research noted around the time of the comprehensive review by Davenport et al. [5] might have been a response to the complex nature of endothelin research, requiring multidisciplinary approaches. This period marked a heightened interest in understanding the multifaceted role of endothelin across different conditions, necessitating diverse expertise.

The development of sparsentan for the treatment of glomerular diseases marked by the works of Rheault et al. [19] and Rovin et al. [6] signifies a major advancement and reflects the field’s growth. This is consistent with the latest surge in research output, indicating the increasing therapeutic applications of endothelin antagonists.

Research into dual endothelin receptor antagonists, such as aprocitentan, highlighted by Schlaich et al. [37], Trensz et al. [38], and the pharmacokinetic and safety profile studies by Sidharta et al. [39] and Gueneau de Mussy et al. [40], has continued to push the boundaries of understanding and treating hypertension and kidney diseases. These studies likely contributed to the sustained upward trend in collaborative research, as comprehensive studies often require the concerted effort of multiple disciplines. The steep increases in author collaboration and the high points in publication volume mirror the field’s active response to new discoveries, clinical trials, and evolving treatment paradigms.

### Web of Science category and journal

A total of 266 journals published ERA in kidney-related articles in 47 Web of Science categories in SCI-EXPANDED. The top 10 productive Web of Science categories are shown in Table 1. The category of pharmacology and pharmacy (277 journals) published the most 231 articles (32% of 713 articles). Compare the top 10 categories in Table 1, 23 articles published in category of transplantation had the highest *APP* of 9.9 authors while pharmacology and pharmacy had an *APP* of 5.7 authors.

**Table 1.** The top 10 most productive Web of Science categories.

Web of Science category	*TP* (%)	No. *J*	*APP*
Pharmacology and pharmacy	231 (32)	277	5.7
Cardiac and cardiovascular systems	154 (22)	142	7.3
Peripheral vascular disease	101 (14)	67	6.6
Respiratory system	86 (12)	65	8.1
Urology and nephrology	70 (10)	88	8.2
Physiology	60 (8.4)	79	6.0
General and internal medicine	41 (5.8)	168	8.2
Research and experimental medicine	40 (5.6)	136	6.4
Critical care medicine	23 (3.2)	35	8.3
Transplantation	23 (3.2)	26	9.9

*TP*: total number of publications; no. *J*: number of journals in a category in 2022; *APP*: average number of authors per publication.

Table 2 displays the top 10 productive journals, along with their *IF*_2022_ and *APP*. Four of the top 10 journals were listed in the Web of Science categories of ‘peripheral vascular disease’ and ‘cardiac and cardiovascular systems,’ respectively. The *Journal of Cardiovascular Pharmacology* (*IF*_2022_ = 3.0) in both the categories of ‘cardiac and cardiovascular systems’ and ‘pharmacology and pharmacy’ published the most 42 articles which represent 5.9% of 713 articles. Compare the top 10 productive journals in Table 2, the *APP* ranged from 9.2 authors in the *Journal of the American Society of Nephrology* to 5.1 authors in the *European Journal of Pharmacology*. According to *IF*_2022_, two journals with *IF*_2022_ more than 100, including the top two journals in 167 journals in the Web of Science category of general and internal medicine: *Lancet* (*IF*_2022_ = 168.9) with five articles and *New England Journal of Medicine* (*IF*_2022_ = 120.7) with two articles.

**Table 2.** The top 10 most productive journals.

Journal	*TP* (%)	*IF*_2022_	*APP*	Web of Science category
*Journal of Cardiovascular Pharmacology*	42 (5.9)	3.0	5.9	Cardiac and cardiovascular systems Pharmacology and pharmacy
*Hypertension*	40 (5.6)	8.3	6.1	Peripheral vascular disease
*European Journal of Pharmacology*	23 (3.2)	5.0	5.1	Pharmacology and pharmacy
*Journal of the American Society of Nephrology*	19 (2.7)	13.6	9.2	Urology and nephrology
*Journal of Hypertension*	16 (2.2)	4.9	7.8	Peripheral vascular disease
*Pulmonary Circulation*	16 (2.2)	2.6	7.4	Cardiac and cardiovascular systems Respiratory system
*Kidney International*	15 (2.1)	19.6	5.9	Urology and nephrology
*American Journal of Physiology-Heart and Circulatory Physiology*	13 (1.8)	4.8	5.7	Cardiac and cardiovascular systems Physiology Peripheral vascular disease
*Circulation*	11 (1.5)	37.8	8.5	Cardiac and cardiovascular systems Peripheral vascular disease
*Pulmonary Pharmacology & Therapeutics*	11 (1.5)	3.2	6.3	Pharmacology and pharmacy Respiratory system

*TP*: total number of publications; %: percentage of articles; *IF*_2022_: journal’s impact factor in 2022; *APP*: average number of authors per publication.

### Publication performance of countries and institutions

Five publication indicators as outlined by Wang et al. [33] were applied to assess and compare the publication performance of various countries and institutions. Among the total of four articles analyzed, tress articles (0.56% of 713 articles) lacked any affiliations in the SCI-EXPANDED databases. The remaining 709 articles were authored by researchers hailing from a diverse range of 49 countries. Notably, 70% of these articles, which amounts to 494 articles, were written by researchers from the same country and represented 34 different countries. In contrast, 30% of the articles (215 articles) resulted from international collaborations, involving authors from 43 countries.

Table 3 presents a comparison of the top 10 productive countries, using five publication indicators by Wang et al. [33]. Five of the top 10 productive countries were in Europe, two in America, two in Asia, and one in Oceania. Egypt with six articles (ranked 25th) was the most productive country in Africa. The USA dominated the five publication indicators with a *TP* of 290 articles (41% of 709 articles), an *IP*_C_ of 171 articles (35% of 494 single-country articles), a *CP*_C_ of 75 articles (35% of 215 internationally collaborative articles), an *FP* of 215 articles (30% of 709 first-author articles), and an *RP* of 217 articles (31% of 704 corresponding-author articles).

**Table 3.** Top 10 productive countries.

Country	*TP*	*TP R* (%)	*IP*_C_*R* (%)	*CP*_C_*R* (%)	*FP R* (%)	*RP R* (%)
USA	290	1 (41)	1 (35)	1 (35)	1 (30)	1 (31)
Germany	120	2 (17)	3 (11)	2 (24)	3 (10)	3 (10)
Japan	98	3 (14)	2 (13)	7 (11)	2 (11)	2 (11)
UK	94	4 (13)	4 (5.9)	3 (22)	5 (6.6)	5 (6.1)
Canada	78	5 (11)	6 (5.7)	5 (13)	4 (7.1)	4 (6.7)
Italy	53	6 (7.5)	7 (3.8)	6 (12)	7 (3.9)	8 (4.1)
Australia	48	7 (6.8)	12 (1.6)	4 (17)	12 (1.7)	10 (2.6)
China	48	7 (6.8)	4 (5.9)	11 (7.9)	6 (4.4)	6 (4.4)
Switzerland	42	9 (5.9)	8 (2.8)	13 (6.5)	7 (3.9)	9 (4.0)
Netherlands	41	10 (5.8)	21 (0.40)	14 (6.0)	7 (3.9)	7 (4.3)

*TP*: total number of articles; *TP R* (%): the percentage of total articles published by each country; *IP*_C_ *R* (%): the rank and percentage of single-country articles in all single-country articles; *CP*_C_ *R* (%): the rank and percentage of internationally collaborative articles in all internationally collaborative articles; *FP R* (%): the rank and percentage of first-author articles in all first-authors articles; *RP R* (%): the rank and percentage of corresponding-author articles in all corresponding-author articles.

Out of the total of 709 articles with affiliations information in the SCI-EXPANDED, 261 articles (37%) were published by single institutions. In contrast, a substantial majority of 448 articles (63%) were the result of inter-institutional collaborations. Table 4 provides an overview of the top 14 productive institutions in ERA in kidney-related research, along with their characteristics [33]. Eight of them were in the USA, two in the UK, and one in Canada, Germany, Mexico, and Netherlands, respectively. The University of Groningen in Netherlands emerged as the leading institution a *TP* of 28 articles (3.9% of 709 articles), a *CP*_I_ of 28 articles (6.3% of 448 inter-institutionally collaborative articles), and an *FP* of 18 articles (2.5% of 709 first-author articles). The University of Groningen had no institutional independent article. Both the University of Edinburgh in the UK and the Michigan State University in the USA ranked the top with an *RP* of 14 articles (2.0% of 699 corresponding-author articles), respectively. Furthermore, the University of Mississippi in the USA with a *TP* of 13 articles (ranked 17th) had the most single-institution articles with an *IP*_I_ of 11 articles (4.2% of 261 single-institution articles).

**Table 4.** Top 14 productive institutions with at least 15 articles.

Institution	*TP*	*TP R* (%)	*IP*_I_*R* (%)	*CP*_I_*R* (%)	*FP R* (%)	*RP R* (%)
University of Groningen, Netherlands	28	1 (3.9)	N/A	1 (6.3)	1 (2.5)	3 (1.9)
Gilead Sciences, Inc., USA	27	2 (3.8)	8 (1.5)	2 (5.1)	9 (1.1)	10 (1.3)
University of Utah, USA	24	3 (3.4)	10 (1.1)	3 (4.7)	19 (0.71)	16 (0.72)
Heidelberg University, Germany	21	4 (3.0)	10 (1.1)	4 (4.0)	6 (1.7)	7 (1.6)
University of Edinburgh, UK	19	5 (2.7)	3 (2.7)	16 (2.7)	2 (2.1)	1 (2.0)
University of Michigan, USA	18	6 (2.5)	N/A	4 (4.0)	52 (0.28)	54 (0.29)
University of Colorado, USA	17	7 (2.4)	40 (0.38)	6 (3.6)	14 (0.85)	16 (0.72)
University of Toronto, Canada	16	8 (2.3)	40 (0.38)	7 (3.3)	116 (0.14)	120 (0.14)
University of Glasgow, UK	15	9 (2.1)	18 (0.77)	12 (2.9)	52 (0.28)	54 (0.29)
National Medical Science and Nutrition Institute Salvador Zubirán, Mexico	15	9 (2.1)	N/A	7 (3.3)	N/A	N/A
Michigan State University, USA	15	9 (2.1)	2 (3.4)	55 (1.3)	3 (1.8)	1 (2.0)
Columbia University, USA	15	9 (2.1)	40 (0.38)	9 (3.1)	34 (0.42)	33 (0.43)
Baylor College of Medicine, USA	15	9 (2.1)	40 (0.38)	9 (3.1)	34 (0.42)	33 (0.43)
Abbott Laboratories, USA	15	9 (2.1)	4 (2.3)	32 (2.0)	12 (1.0)	12 (1.0)

*TP*: total number of articles; *TP R* (%): the percentage of total articles published by each institution; *IP*_I_ *R* (%): the rank and percentage of single- institution publications in all single-institution articles; *CP*_I_ *R* (%): the rank and percentage of inter-institutionally collaborative articles in all inter-institutionally collaborative articles; *FP R* (%): the rank and percentage of first-author articles in all first-authors articles; *RP R* (%): the rank and percentage of corresponding-author articles in all corresponding-author articles; N/A: data are not available.

### Publication performances: authors

For ERA in kidney articles, the *APP* was 7.0 authors whereas the maximum number of authors was 33 in one article. Of the 710 articles with author information in the SCI-EXPANDED, 64% articles were published by groups of three to eight authors, including 84 articles (12% of 710 articles), 80 (11%), 79 (11%), 76 (11%), 75 (11%), and 65 (9.2%) were written by groups of 5, 4, 6, 7, 3, and 8 authors, respectively. Table 5 lists the top 11 productive authors with 14 articles or more. H.J.L. Heerspink dominated all the three publication indicators with a *TP* of 30 articles, an *FP* of nine articles, and an *RP* of 22 articles. All the top 11 productive authors had no single-author article. V. Perkovic, H.H. Parving, R. Correa-Rotter, H. Makino, and C. Blair did not have any first-author articles or corresponding-author articles. Only two of the 11 productive authors including H.J.L. Heerspink and D.M. Pollock were found to be the top 11 publication potential authors as evaluated by *Y*-index.

**Table 5.** Top 11 productive authors with 14 articles or more.

Author	Rank (*TP*)	Rank (*FP*)	Rank (*RP*)	*h*	Rank (*j*)
H.J.L. Heerspink	1 (30)	1 (9)	1 (22)	1.182	1 (31)
D. De Zeeuw	2 (22)	90 (1)	37 (2)	1.107	62 (3)
V. Perkovic	3 (21)	N/A	N/A	0	766 (0)
D.E. Kohan	4 (20)	12 (3)	22 (3)	π/4	14 (6)
D.J. Webb	5 (19)	90 (1)	9 (5)	1.373	14 (6)
D.M. Pollock	6 (18)	6 (4)	3 (12)	1.326	3 (15)
H.H. Parving	7 (17)	N/A	N/A	0	766 (0)
R. Correa-Rotter	7 (17)	N/A	N/A	0	766 (0)
H. Makino	9 (15)	N/A	N/A	0	766 (0)
R.J. Oudiz	10 (14)	28 (2)	37 (2)	π/4	28 (4)
C. Blair	10 (14)	N/A	N/A	0	766 (0)

*TP*: total number of highly cited articles; *FP*: number of first-author articles; *RP*: number of corresponding-author articles; *j*: a *Y*-index constant related to the publication potential; *h*: a *Y*-index constant related to the publication characteristics; N/A: not available.

In the total of 686 articles (97% of 710 articles) had both first and corresponding authors information in SCI-EXPANDED, were extensively investigated based on the *Y*-index. The 686 articles were contributed by 3151 authors in which 2386 authors (76% of 3151 authors) had no first- and corresponding-author articles with *Y*-index (0, 0); 207 authors (6.6%) authors published only corresponding-author articles with *h* = π/2; 33 (1.0%) authors published more corresponding-author articles than first-author articles with π/2 > *h* > π/4 (*FP* > 0); 242 authors (7.7%) authors published the same number of first- and corresponding-author articles with *h* = π/4 (*FP* > 0 and *RP* > 0); eight authors (0.25%) authors published more first-author articles than corresponding-author articles with π/4 > *h* > 0 (*RP* > 0); and 275 authors (8.7%) authors published only first-author articles with *h* = 0. Highly percentage of authors without first- and corresponding-author articles in medical-related topics were also reported in Q fever (76%) [27], and bruxism (70%) [41].

**Figure 2.**
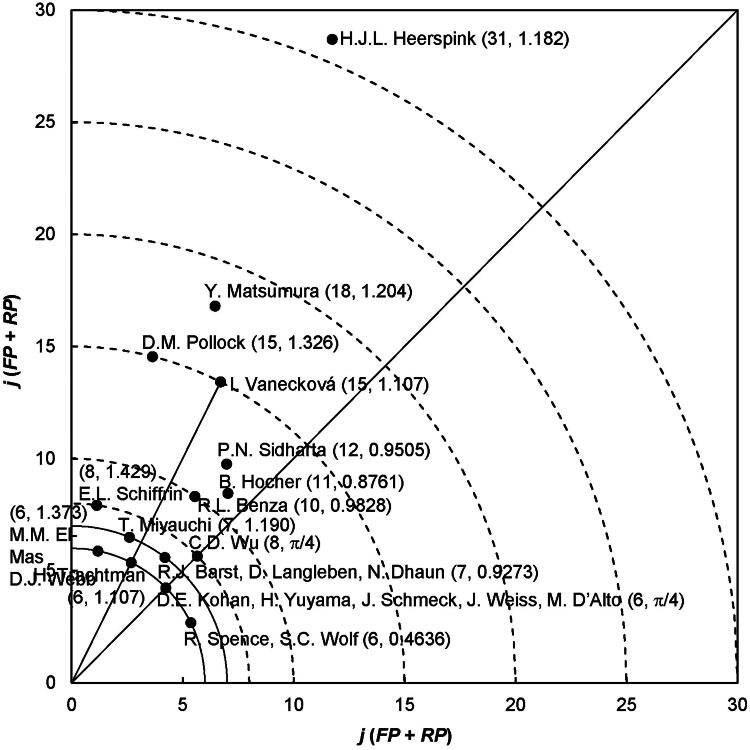
Top 23 authors with *Y*-index (*j* ≥ 6).

In the polar coordinate system (Figure 2), the distribution of *Y*-index values (*j*, *h*) was used to illustrate the performance of the top 23 potential authors in ERA in kidney articles, specifically those with a *j* ≥ 6 articles. Each point on the graph corresponds to a *Y*-index coordinate (*j*, *h*), representing either a single author or multiple authors. For instance, authors like D.J. Webb and M.M. El-Mas were located at *Y*-index (6, 1.373). H.J.L. Heerspink, with a *Y*-index (31, 1.182), exhibited the highest publication potential among all authors. Following was Y. Matsumura, with a *Y*-index of (18, 1.204). It was worth noting that Heerspink’s remarkable performance was particularly surprising because Heerspink had published significantly more articles as a corresponding author than as a first author, with an *h*-value of 1.182. This indicates that Heerspink is still actively involved in research and continues to make significant contributions to the field. However, it was worth noting that authors with the highest publication potential in articles, as indicated by *h*-values of 1435, 0.9595, 0.8652, and π/4 were found in Q fever [27], temporomandibular disorders [28], bruxism [41], and fracture nonunion [42], respectively.

D.J. Webb and M.M. El-Mas (6, 1.373), H. Trachtman (6, 1.107), D.E. Kohan, H. Yuyama, J. Schmeck, J. Weiss, and M. D’Alto (6, π/4), R. Spence and S.C. Wolf (6, 0.4636) each with a *j*-value of six articles, were positioned on the same curve (*j* = 6) in Figure 2. This suggests that they possessed the same level of publication potential in articles. However, their publication characteristics, as highlighted by Ho and Hartley [43], differed significantly. Webb and El-Mas had the highest *h*-value of 1.373 indicating that they had the highest ratio of corresponding-author articles to first-author articles, followed by Trachtman with an *h*-value of 1.107. These differences in publication characteristics suggest that Webb and El-Mas were more involved in supervision. Kohan, Yuyama, Schmeck, Weiss, and D’Alto (6, π/4) published the same number of the first-author articles and the corresponding-author articles with an *h*-value of π/4. In contrast, Spence and Wolf had an *h*-value of 0.4636, indicating that Spence and Wolf published more first-author articles than corresponding-author articles. This suggests active involvement as a primary author in their research projects. Similarly, T. Miyauchi (7, 1.190) and R.J. Barst, D. Langleben, and N. Dhaun (7, 0.9273); E.L. Schiffrin (8, 1.429) and C.D. Wu (8, π/4); and D.M. Pollock (15, 1.326) and I. Vanecková (15, 1.107) each with a *j*-value of 7, 8, and 15 articles, respectively, were positioned on the same curve (*j* = 7, 8, and 15) in Figure 2.

Authors such as C.D. Wu (8, π/4) and D.E. Kohan, H. Yuyama, J. Schmeck, J. Weiss, and M. D’Alto (6, π/4) were all positioned along the diagonal line (*h* = π/4) in Figure 2. This alignment signifies that they shared the same publication characteristics, but their publication potential varied. Wu had the higher publication potential, boasting a *j*-value of eight articles, indicating a substantial number of articles. Following were Kohan, Yuyama, Schmeck, Weiss, and D’Alto with a *j*-value of six articles. While these authors shared the same publication characteristics with a *h*-value of π/4, including a combination of supervision and active research participation, their publication potential varied, with Wu being the most prolific. This could indicate that Wu is a seasoned senior researcher. In contrast, authors like Kohan and the four others might have less research experience but still make valuable contributions to the field. Similarly, I. Vanecková (15, 1.107) and H. Trachtman (6, 1.107) also located along the straight line (*h* = 1.107) in Figure 2. They have the same publication characteristics with a *h*-value of 1.107. However, Vanecková had higher publication potential with a *j*-value of 15 articles. The positioning of authors on the graph, either along specific curves or lines originating from the origin, represents distinct author publication potential or publication characteristics within various families [27]. It was essential to acknowledge that a potential source of bias in the analysis of authorship can be attributed to several factors, including different authors sharing the same name or the same author using different names over time, as highlighted by Chiu and Ho [31]. These factors can lead to challenges in accurately attributing research contributions and understanding the dynamics of authorship within a field.

### Research focuses and their development trends

In the last decade, researchers have proposed using the distribution of words in article titles and abstracts, author keywords, and *Keywords Plus* to identify the main research focuses and their trends in a research field [44,45]. The word analysis results were as word bank to be used to find research focused. Excepted the search keywords, the 20 most frequently used word in title and author keywords are listed in Table 6.

**Table 6.** The 20 most frequently used words in article title and author keywords excepted search keywords.

Word in title	*TP*	Rank (%)	Author keywords	*TP*	Rank (%)
Pulmonary	252	1 (35)	Endothelin	135	1 (24)
Receptor	243	2 (34)	Pulmonary hypertension	79	2 (14)
Endothelin	230	3 (32)	Pulmonary arterial hypertension	76	3 (14)
Arterial	152	4 (21)	Endothelin-1	71	4 (13)
Rats	132	5 (19)	Endothelin receptor antagonist	51	5 (9.1)
Antagonist	117	6 (16)	Bosentan	32	6 (5.7)
Eta	85	7 (12)	Endothelin receptor antagonists	32	6 (5.7)
Patients	81	8 (11)	Nitric oxide	28	8 (5.0)
Endothelin-1	75	9 (11)	Endothelin receptors	24	9 (4.3)
Effects	68	10 (10)	Blood pressure	22	10 (3.9)
Blockade	66	11 (9.3)	Chronic kidney disease	20	11 (3.6)
Treatment	62	12 (8.7)	Heart failure	18	12 (3.2)
Renal	59	13 (8.3)	Tadalafil	17	13 (3.0)
Antagonists	56	14 (7.9)	Angiotensin ii	15	14 (2.7)
Chronic	49	15 (6.9)	Bq-123	15	14 (2.7)
Vascular	49	15 (6.9)	Eta receptor	15	14 (2.7)
Selective	45	17 (6.3)	Endothelin receptor	14	17 (2.5)
Therapy	45	17 (6.3)	Combination therapy	13	18 (2.3)
Disease	44	19 (6.2)	Endothelin antagonist	13	18 (2.3)
Rat	39	20 (5.5)	Sildenafil	13	18 (2.3)

*TP*: total number of publications; %: percentage of articles; N/A: data is not available.

The examination of prevalent words in ERA research highlights the evolving scientific dialogue in this area. A notable concentration of terms related to pulmonary health – such as ‘pulmonary,’ ‘arterial,’ and specific diagnoses like PH and PAH – underscores the dominant focus on respiratory diseases. This emphasis highlights the critical role of ERAs in managing these conditions and the broader impact of endothelin blockade in vascular-related pathologies. However, recent studies have expanded beyond pulmonary applications, demonstrating increasing interest in the role of ERAs in CKD, glomerular diseases, and diabetic nephropathy (DN).

The recurrent mention of ‘endothelin’ and its associated terms in scholarly articles indicates the essential role of the endothelin signaling pathway in heart and kidney diseases. The distinction made between different components of this pathway, including ‘endothelin-1,’ ‘endothelin receptor antagonist,’ and ‘endothelin receptors,’ reflects a deep dive into this complex biological system. This level of detail suggests a concerted effort to grasp how the endothelin pathway contributes to disease processes and how it might be effectively targeted with therapies.

#### Clinical applications in kidney diseases

Recent research has investigated ERA therapy as a strategy to slow CKD progression, particularly in proteinuric CKD, by addressing persistent ET-1-mediated damage. Renal ET-1 levels are elevated in various CKD etiologies, including diabetic, hypertensive, and glomerular diseases, and correlate with disease severity (Table 7) [6,14,15,19,20,37,46–78]. Blocking ETA has been shown to mitigate key pathogenic processes, such as glomerulosclerosis and tubulointerstitial fibrosis. Preclinical studies indicate that chronic ETA receptor blockade reduces kidney inflammation, fibrosis, and proteinuria. For instance, transgenic mice overexpressing ET-1 develop glomerulosclerosis and interstitial fibrosis – both of which are alleviated by ERA treatment.

**Table 7.** Summary of endothelin receptor antagonists and their applications.

Drug	Receptor selectivity	Indications	Key effects	Adverse effects
Sparsentan	Dual ETA/angiotensin II	IgAN, FSGS, Alport syndrome	Reduces proteinuria, preserves kidney function	Hypotension, edema, hyperkalemia
Atrasentan	ETA-selective	DKD, IgAN, hypertensive nephropathy	Reduces proteinuria, upregulates klotho, reduces fibrosis	Edema, anemia, weight gain
Zibotentan	ETA-selective	CKD, IgAN (with dapagliflozin)	Reduces proteinuria, improves eGFR	Fluid retention at high doses
SC0062	ETA-selective	IgAN	Reduces proteinuria (51.6% at 20 mg dose), low edema incidence	Not yet reported
Aprocitentan	Dual ETA/ETB	Resistant hypertension	Lowers blood pressure	Fluid retention
Ambrisentan	ETA-selective	FSGS	Reduces proteinuria, protects podocytes	Not reported
Avosentan	ETA-selective	DKD, hypertensive nephropathy	Reduces proteinuria, improves renal inflammation	Fluid overload, CHF risk
Darusentan	ETA-selective	Polycystic kidney disease	Increases tubular cell proliferation	Fluid retention
Bosentan	Dual ETA/ETB	Hypertensive nephropathy, polycystic kidney disease	Limited albuminuria reduction	Renal blood flow reduction
BQ-788	ETB-selective	Diabetic nephropathy	Reduces renal blood flow, decreases GFR	Not reported

AKI: acute kidney injury; APP: average number of authors per publication; BP: blood pressure; CHF: congestive heart failure; CKD: chronic kidney disease; DKD: diabetic kidney disease; eGFR: estimated glomerular filtration rate; ERA: endothelin receptor antagonist; ET-1: endothelin-1; ETA: endothelin A receptor; ETB: endothelin B receptor; FSGS: focal segmental glomerulosclerosis; GFR: glomerular filtration rate; HTN: hypertension; IgAN: IgA nephropathy; KD: kidney disease; PAH: pulmonary arterial hypertension; PKD: polycystic kidney disease; RAAS: renin–angiotensin–aldosterone system; RCT: randomized controlled trial; SCI-EXPANDED: Science Citation Index Expanded; Zib: zibotentan.

Small clinical studies suggest that selective ETA blockers significantly reduce blood pressure, arterial stiffness, and proteinuria, thus improving CKD progression risk factors. However, due to safety concerns, ERAs remain investigational for routine CKD therapy, with use generally limited to clinical trials or specific conditions, such as PH (Table 8) [6,14,15,19,20,37,46–78].

**Table 8.** Endothelin receptor antagonists in kidney and cardiovascular diseases [6,15, 19,20, 37,46, 48,49, 54–56,59, 64–78].

Disease/condition	Key pathophysiology of ET-1	ERA(s) used	Key findings	Notable clinical trials
DKD	ET-1 promotes inflammation, fibrosis, vasoconstriction, and proteinuria	Atrasentan, avosentan, zibotentan	Atrasentan reduced primary kidney outcomes by 35% in SONAR trial; combination with SGLT2 inhibitors enhances kidney protection	SONAR, ZENITH-HP
IgAN	ET-1 contributes to mesangial cell proliferation, podocyte injury, and proteinuria	Sparsentan, atrasentan, SC0062	Sparsentan reduced proteinuria by 35% vs. irbesartan; SC0062 reduced proteinuria by 51.6% at 20 mg dose	PROTECT, 2-SUCEED
FSGS	ET-1 leads to podocyte loss, proteinuria, and glomerulosclerosis	Sparsentan, ambrisentan	Sparsentan attenuated podocyte injury and reduced proteinuria	SPARTAN
Alport syndrome	ET-1 contributes to glomerular basement membrane thickening and interstitial fibrosis	Sparsentan, atrasentan, sitaxsentan	Sparsentan delayed GFR decline and mitigated hearing loss progression based on animal studies	CARDINAL, JASMIN
Hypertensive nephropathy	ET-1 causes endothelial dysfunction, increased blood pressure, and proteinuria	Atrasentan, avosentan, zibotentan	Combination of ERAs with ARBs improved renal function and proteinuria	ZENITH-HP, ASCEND
Treatment-resistant hypertension	ET-1 drives systemic vasoconstriction and vascular remodeling	Aprocitentan	Aprocitentan significantly reduced blood pressure in treatment-resistant hypertension	PRECISION
CKD secondary to systemic sclerosis	ET-1 contributes to renal vasculopathy and fibrosis	Zibotentan	Improved eGFR and reduced proteinuria when combined with SGLT2 inhibitors	ZENITH-CKD
PKD	ET-1 promotes tubular cyst growth and inflammation	Darusentan, bosentan	ERAs increased tubular cell proliferation but were associated with fluid retention	PKD-ERA study
AKI	ET-1 exacerbates endothelial dysfunction, tubular injury, and hypoxia	Sitaxsentan	Sitaxsentan reversed endothelial dysfunction and reduced AKI progression	AKI-ERA
Heart failure with CKD	ET-1 increases vascular resistance and fluid retention, worsening HF outcomes	Atrasentan, zibotentan	Selective ETA antagonists improved kidney function and reduced fluid overload in HF patients	ZENITH-HF

AKI: acute kidney injury; ARB: angiotensin receptor blocker; ASCEND: Avosentan in Diabetic Nephropathy Trial; BP: blood pressure; CARDINAL: Study Evaluating Sparsentan in Alport Syndrome; CKD: chronic kidney disease; ERA: endothelin receptor antagonist; ET-1: endothelin-1; ETA: endothelin A receptor; ETB: endothelin B receptor; eGFR: estimated glomerular filtration rate; FSGS: focal segmental glomerulosclerosis; GFR: glomerular filtration rate; HTN: hypertension; IgAN: IgA nephropathy; JASMIN: Study Evaluating Sitaxsentan in Alport Syndrome; KD: kidney disease; PAH: pulmonary arterial hypertension; PKD: polycystic kidney disease; PRECISION: Aprocitentan for Resistant Hypertension Trial; PROTECT: Sparsentan for IgA Nephropathy Trial; RAAS: renin–angiotensin–aldosterone system; RCT: randomized controlled trial; SCI-EXPANDED: Science Citation Index Expanded; SGLT2: sodium-glucose cotransporter-2; SONAR: Study of Atrasentan in Diabetic Nephropathy; SPARTAN: Sparsentan in FSGS Trial; 2-SUCEED: SC0062 for IgA Nephropathy Trial; ZENITH-CKD: Zibotentan for CKD Trial; ZENITH-HF: Zibotentan in Heart Failure Trial; ZENITH-HP: Zibotentan in Hypertensive Nephropathy Trial.

##### Diabetic nephropathy (diabetic kidney disease)

Diabetic nephropathy exemplifies CKD where the endothelin system is overactive. Hyperglycemia, insulin resistance, and oxidative stress in diabetes elevate renal ET-1 levels, contributing to glomerular hyperfiltration, podocyte injury, and proteinuria. ERAs have been extensively studied in DN, with early trials showing promise but also revealing challenges.

For example, the selective ETA antagonist avosentan significantly reduced albuminuria when added to standard therapy, but the Phase 3 ASCEND trial was terminated early due to a higher incidence of cardiovascular events, particularly fluid overload and congestive heart failure. This identified fluid retention as a major limiting side effect in diabetic patients. Consequently, attention turned to atrasentan, a highly selective ETA antagonist. In smaller trials, low-dose atrasentan significantly reduced albuminuria while managing edema through careful monitoring.

The Phase 3 SONAR trial evaluated atrasentan’s long-term renal outcomes in DN, utilizing an ‘enrichment’ design in which only patients who responded well to atrasentan without significant edema were randomized. Over a median follow-up of 2.2 years, atrasentan therapy led to a 35% reduction in renal events, confirming its nephroprotective effects. However, atrasentan has not yet obtained regulatory approval for DN, partly due to early termination of the SONAR trial. Nonetheless, these studies suggest that ERAs can meaningfully delay CKD progression in carefully selected DN patients.

##### Glomerular diseases (IgA nephropathy, FSGS)

Endothelin has also emerged as a therapeutic target in non-diabetic glomerular diseases characterized by proteinuria. IgA nephropathy and FSGS have been active areas of ERA research, as these diseases often exhibit persistent proteinuria despite RAAS blockade.

In IgAN, the dual endothelin/angiotensin receptor antagonist sparsentan demonstrated superior proteinuria reduction compared to standard therapy in the Phase 3 PROTECT trial. Based on these findings, sparsentan received FDA approval in 2023 as the first ERA-based therapy for IgAN. The ALIGN trial is currently evaluating atrasentan in IgAN, with interim results showing significant proteinuria reductions.

In FSGS, sparsentan demonstrated superior proteinuria reduction in the Phase 2 DUET trial compared to irbesartan. The larger Phase 3 DUPLEX trial is evaluating long-term renal outcomes, with early findings suggesting that sparsentan not only lowers proteinuria but may also slow disease progression. These results position ERAs as promising therapeutic options for proteinuric glomerular diseases.

#### Comparison of ERAs

ERAs differ in receptor selectivity and pharmacologic properties [6,14,15,19,20,37,46–78]:

- *Bosentan*: A nonselective ETA/ETB blocker with a high risk of hepatotoxicity, limiting its use in kidney disease.
- *Ambrisentan*: A highly ETA-selective antagonist with a lower risk of liver toxicity, though fluid retention remains a concern.
- *Macitentan*: A dual ERA with improved tissue penetration and fewer side effects than bosentan.
- *Atrasentan*: A highly selective ETA antagonist with strong efficacy in DN and IgAN trials.
- *Sparsentan*: A dual ETA/AT1 receptor antagonist that combines antiproteinuric mechanisms and was recently approved for IgAN.

#### Safety and adverse effects

Despite their therapeutic potential, ERAs are associated with notable side effects [6,14,15,19,20,37,46–78]:

- *Fluid retention and edema*: The most common adverse effect, ranging from mild peripheral edema to heart failure exacerbation. Diuretics and dietary sodium restriction are often used to mitigate this risk.
- *Cardiovascular effects*: ERAs can lower blood pressure but may cause hypotension, reflex tachycardia, and nasal congestion.
- *Hepatotoxicity*: Bosentan is linked to dose-dependent liver enzyme elevations, whereas newer ERAs pose a lower hepatic risk.
- *Hematologic effects*: ERAs may cause mild anemia due to hemodilution.
- *Teratogenicity*: ERAs are contraindicated in pregnancy due to fetal malformation risks.

#### Regulatory status and ongoing research

Historically, ERAs were approved for PAH. However, in 2023, the FDA granted accelerated approval for sparsentan in IgAN, marking the first ERA-based therapy in nephrology. Ongoing research aims to optimize dosing strategies, mitigate edema risks by combining ERAs with SGLT2 inhibitors, and expand indications to other glomerular diseases.

##### Integration into research trends

Discussions around specific pharmaceuticals such as ‘bosentan,’ ‘tadalafil,’ and ‘sildenafil,’ along with ‘nitric oxide’ and ‘angiotensin II,’ highlight the diverse therapeutic agents under investigation. This variety showcases the collaborative nature of research in this field, merging insights from pharmacology, cardiology, and nephrology to pioneer novel treatment avenues. The exploration of conditions like ‘chronic kidney disease’ and ‘heart failure,’ alongside treatment methods such as ‘combination therapy’ and ‘blockade,’ reveals an extensive investigational reach. This broad approach signals a keen interest in unraveling disease mechanisms, refining treatment protocols, and discovering combined therapies to improve patient care.

Additionally, the analysis points to an expansion of research interests, extending beyond pulmonary issues to include a broader spectrum of diseases and therapeutic tactics. This progression may encourage further studies on the utility of ERAs in treating systemic conditions beyond their current applications, thus broadening the research and treatment landscape. The data also suggest ample opportunity for cross-disciplinary research, especially in fully understanding the endothelin pathway’s role in various diseases. Collaborative efforts could lead to groundbreaking discoveries and therapeutic innovations, offering a richer, more comprehensive view of potential treatment modalities.

In the years 2022–2023, there has been a noticeable shift in the research focus toward hypertension and glomerular diseases, as evidenced by key published clinical trials that have explored innovative therapeutic approaches [6,19,37,75]. These studies have significantly contributed to the expanding body of knowledge on the treatment of these conditions, highlighting the evolving landscape of cardiovascular and renal disease management. Among these pivotal contributions is the study conducted by Rheault et al. [19], published in ‘The New England Journal of Medicine’ in 2023. This research examined the effectiveness of sparsentan versus irbesartan in treating FSGS, a condition that often leads to ESKD due to the lack of effective treatment options. In the final analysis at week 108 of the study, the DUPLEX trial observed no significant difference in the estimated glomerular filtration rate (eGFR) between sparsentan and irbesartan treatments [19]. This outcome suggests that while sparsentan offers a superior reduction in proteinuria compared to irbesartan, both treatments exhibit comparable effects on kidney function over the course of the trial. Additionally, the PROTECT study by Rovin et al. [6], also published in ‘The Lancet,’ investigated the efficacy and safety of sparsentan versus irbesartan in patients with IgAN over a 2-year period. The trial’s findings underscored sparsentan’s superior performance in slowing the progression of IgAN, marking a significant advancement in the treatment of this common glomerular disease.

### Gender distribution in authorship

The evolving role of female researchers in the field of ERA research warrants attention, given the broader discussions on gender disparities in academic medicine. To assess this, we analyzed the gender distribution of authors in the 713 identified ERA-related publications. Using publicly available name-based gender classification databases, we assigned probable gender identities to first and corresponding authors, acknowledging inherent limitations in such methodology. Our findings indicate that while male authors have historically dominated the field, the proportion of female first and corresponding authors has gradually increased over the past two decades. Specifically, female first authorship increased from approximately 12% in the 1990s to 28% in the last five years, while female corresponding authorship rose from 10% to 24% during the same period. Notably, interdisciplinary collaborations and multi-author studies showed a higher representation of female contributors, suggesting a growing integration of women in leadership roles within this research domain. This trend aligns with broader efforts in nephrology and cardiovascular research to promote gender equity in authorship and leadership positions. Nonetheless, despite this positive trajectory, female representation in senior authorship remains lower than expected, highlighting the need for continued efforts to support gender diversity in ERA research.

### Regional distribution of authorship

Understanding the geographic distribution of authorship is essential for assessing global contributions to ERA research. We examined the regional affiliations of first and corresponding authors in the 713 identified ERA-related publications. Our findings indicate that while ERA studies have been conducted worldwide, there are notable disparities in authorship trends across different regions. Specifically, North America (led by the United States) and Europe (notably the Netherlands, Germany, and the UK) accounted for the majority of corresponding author affiliations, with the United States alone contributing 31% of all corresponding author articles. In contrast, while a significant number of ERA-related studies focused on populations in Asia, only 14% of corresponding authors were affiliated with Asian institutions. A similar trend was observed in Africa and South America, where research contributions were often linked to international collaborations rather than being led by local investigators.

Further analysis revealed that studies conducted in Asian populations were frequently coauthored by researchers from European or North American institutions, suggesting strong international collaborations but also indicating potential disparities in local research leadership. The University of Groningen (Netherlands) and leading institutions in the United States frequently collaborated on studies based in Asia and other regions. While such collaborations contribute to scientific advancement, they also highlight the need for strengthening local research capacity and leadership in Asia, Africa, and South America to ensure equitable representation in the global ERA research landscape. Moving forward, efforts to support local investigator-led research in these regions may help balance global contributions to ERA studies and improve representation in leading nephrology and cardiovascular journals.

### Clinical applications of endothelin A receptor antagonists in kidney diseases and comparison with other therapies

The therapeutic role of ERAs in kidney disease has expanded significantly, with growing evidence supporting their efficacy across various renal pathologies. Initially developed for PAH, ERAs such as atrasentan, sparsentan, and aprocitentan have demonstrated beneficial effects in CKD and glomerular disorders. Clinical trials have shown that ERAs effectively reduce proteinuria and slow kidney function decline, particularly in proteinuric conditions such as FSGS and IgAN. For example, the PROTECT trial demonstrated that sparsentan provided superior reductions in proteinuria compared to irbesartan in patients with IgAN, while the SONAR trial showed that atrasentan reduced albuminuria and slowed CKD progression in patients with type 2 diabetes.

**Figure 3.**
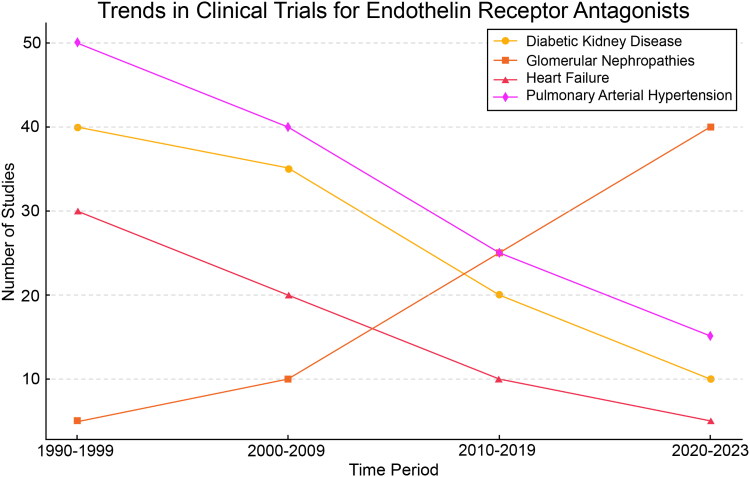
Trends in clinical trials for endothelin receptor antagonists (ERAs) over time. This figure illustrates the evolution of ERA-related clinical trials across different disease categories from 1990 to 2023. The trends reflect a shift in research priorities, with a growing emphasis on ERA applications in GN within nephrology.

When compared to RAS inhibitors, ERAs provide additional proteinuria-lowering effects through distinct mechanisms, particularly by mitigating endothelin-induced inflammation, vasoconstriction, and fibrosis. However, ERA use has been limited by concerns regarding fluid retention and associated adverse effects. Ongoing research aims to optimize the balance between efficacy and safety, including strategies such as dual ERA-SGLT2 inhibitor therapy, which has shown promise in reducing fluid retention while preserving the renoprotective benefits of ERAs. Further comparative effectiveness studies are warranted to determine the optimal therapeutic positioning of ERAs relative to standard-of-care treatments in nephrology (Figure 3).

### Role of multidisciplinary collaboration in endothelin A receptor antagonist research

The advancement of ERA research in kidney disease has been driven by multidisciplinary collaboration between nephrologists, cardiologists, pharmacologists, and basic scientists. Translational research has played a crucial role in elucidating the mechanisms by which endothelin signaling contributes to CKD progression, glomerular injury, and hypertension. For instance, preclinical studies have provided critical insights into the role of ET-1 in podocyte dysfunction and glomerular permeability, paving the way for targeted ERA trials in glomerular diseases.

Clinical research has also benefited from strong interdisciplinary collaboration, particularly in large-scale trials evaluating ERAs for kidney disease. The integration of pharmacokinetics, biomarker discovery, and precision medicine approaches has enhanced our understanding of patient subgroups that may derive the greatest benefit from ERAs. Future research should continue to foster collaboration between basic and clinical sciences, with an emphasis on biomarker-driven trial designs and mechanistic studies that bridge the gap between experimental models and clinical practice.

### Future directions in endothelin A receptor antagonist research

Looking ahead, several key areas warrant further investigation in ERA research:

1. *Development of next-generation ERAs*:
  There is an ongoing need to develop novel ERAs with improved selectivity and reduced adverse effects. Strategies such as biased endothelin receptor agonism, which selectively modulates downstream signaling pathways while minimizing fluid retention, hold promise for enhancing ERA safety profiles. Additionally, combination therapies that pair ERAs with SGLT2 inhibitors, mineralocorticoid receptor antagonists, or other nephroprotective agents should be explored to optimize efficacy.
2. *Design of future clinical trials*:
  Future trials should focus on refining the patient selection criteria for ERA therapy, particularly in subgroups such as patients with non-diabetic glomerular diseases, hypertensive nephropathy, and treatment-resistant CKD. Adaptive trial designs incorporating precision medicine approaches, including the use of genetic and proteomic markers to identify responders, may enhance therapeutic targeting. Additionally, long-term outcome studies are needed to evaluate the durability of ERA benefits and their impact on major renal endpoints, such as progression to ESKD and dialysis initiation.
3. *Exploring ERA use beyond traditional indications*:
  Beyond CKD and PAH, emerging evidence suggests potential applications of ERAs in conditions such as lupus nephritis, cardiorenal syndromes, and resistant hypertension. Investigating the role of endothelin signaling in these conditions may uncover novel therapeutic avenues for ERA use. By addressing these future directions, ERA research can continue to evolve and contribute to innovative treatment strategies in nephrology and beyond.

## Conclusions

ETA receptor antagonists, particularly regarding kidney health, is seeing a surge in collaborative and international efforts aimed at improving both the understanding of these conditions and how they are treated. The United States is leading the way, with its researchers and institutions making notable contributions that are driving the field forward. This collaborative spirit is setting the stage for a future where the study of ERAs is both vigorous and innovative.

There is a growing emphasis on exploring specific drugs and the idea of using more than one treatment together, reflecting the ongoing search for treatments that are not only more effective but also customized to meet individual patient needs. This includes a keen interest in developing new drugs, indicating a significant opportunity for the creation of next-generation ERAs. These future drugs aim to be more effective, have fewer side effects, and be easier for patients to consistently take. Such developments suggest that the field is on the brink of major breakthroughs that could significantly change how patient care is approached, thanks to new treatment options and a deeper grasp of the underlying disease processes.

## Data Availability

All data that support this study has been provided and are also available on request from the corresponding author.
